# Human Pluripotent Stem Cells Derived Endothelial Cells Repair Choroidal Ischemia

**DOI:** 10.1002/advs.202302940

**Published:** 2023-12-19

**Authors:** Mengda Li, Peiliang Wang, Si Tong Huo, Hui Qiu, Chendi Li, Siyong Lin, Libin Guo, Yicong Ji, Yonglin Zhu, Jinyang Liu, Jianying Guo, Jie Na, Yuntao Hu

**Affiliations:** ^1^ Eye Center Beijing Tsinghua Changgung Hospital Beijing 102218 China; ^2^ Institute for Precision Medicine Tsinghua University Beijing 100084 China; ^3^ School of Clinical Medicine Tsinghua University Beijing 100084 China; ^4^ SXMU‐Tsinghua Collaborative Innovation Center for Frontier Medicine School of Medicine Tsinghua University Beijing 100084 China; ^5^ State Key Laboratory for Complex, Severe, and Rare Diseases Tsinghua University Beijing 100084 China; ^6^ Center for Stem Cell Biology and Regenerative Medicine School of Medicine Tsinghua University Beijing 100084 China; ^7^ School of Life Sciences Tsinghua University Beijing 100084 China; ^8^ Center for Reproductive Medicine Department of Obstetrics and Gynaecology Peking University Third Hospital Beijing 100191 China

**Keywords:** choroidal ischemia, endothelial cells, human pluripotent stem cells, ischemia repair, vessel regeneration

## Abstract

Choroidal atrophy is a common fundus pathological change closely related to the development of age‐related macular degeneration (AMD), retinitis pigmentosa, and pathological myopia. Studies suggest that choroidal endothelial cells (CECs) that form the choriocapillaris vessels are the first cells lost in choroidal atrophy. It is found that endothelial cells derived from human pluripotent stem cells (hPSC‐ECs) through the *MESP1+* mesodermal progenitor stage express CECs‐specific markers and can integrate into choriocapillaris. Single‐cell RNA‐seq (scRNA‐seq) studies show that hPSC‐ECs upregulate angiogenesis and immune‐modulatory and neural protective genes after interacting with ex vivo ischemic choroid. In a rat model of choroidal ischemia (CI), transplantation of hPSC‐ECs into the suprachoroidal space increases choroid thickness and vasculature density. Close‐up examination shows that engrafted hPSC‐ECs integrate with all layers of rat choroidal vessels and last 90 days. Remarkably, EC transplantation improves the visual function of CI rats. The work demonstrates that hPSC‐ECs can be used to repair choroidal ischemia in the animal model, which may lead to a new therapy to alleviate choroidal atrophy implicated in dry AMD, pathological myopia, and other ocular diseases.

## Graphical abstract

Choroid ischemia is implicated in many blinding eye diseases. The study differentiates human pluripotent stem cells into choroid‐like endothelial cells and transplants them into an animal model of Choroid ischemia. Engrafted endothelial cells repair choroid vasculature and improve the visual function of CI animals. This research establishes a promising new cellular therapy to treat eye diseases linked to choroidal ischemia.

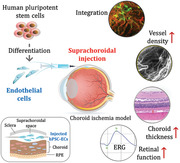

## 1 Introduction

The delicate blood vessel system supplying the nutrients and oxygen to the retina is crucial for its well‐being and the generation of our vision.^[^ ^1^ ^]^ The central retinal artery supplies the inner retina, and the choriocapillaris vessels nourish the outer retina.^[^ ^2^ ^]^ The choroidal blood supply accounts for 85% of the blood flow to the eye.^[^ ^3^ ^]^ Besides contributing to the outer retinal circulation and maintaining the blood‐retina barrier, the choroid also acts as a heat sink to thermally protect the retina.^[^ ^4^, ^5^ ^]^ The choroid consists of four layers, from the outermost to the innermost: suprachoroidea, large‐vessel layer, intermediate‐vessel layer, and choriocapillaris.^[^ ^6^, ^7^ ^]^ The choriocapillaris is a highly anastomosed network covering the Bruch's membrane which is basal to the retinal pigmented epithelium (RPE) layer (Figure 2B). The metabolite and oxygen exchange between RPE and choroid is vital for RPE survival.^[^ ^5^ ^]^ Loss of RPE cells is often coupled with atrophic choriocapillaris, while choroid degeneration could be found independent of RPE loss.^[^ ^8^ ^]^ Choroid thinning is a hallmark of the pathological change of the choroid. It is implicated in immature choroid neovascularization and other neurodegenerative diseases.^[^ ^7^, ^9^, ^10^ ^]^ The choriocapillaris are prone to inflammations, which would cause uveitis and punctate inner choroidopathy.^[^ ^11^, ^12^ ^]^ Genetically inherited disorders include choroideremia, Retinitis Pigmentosa,^[^ ^13^, ^14^ ^]^ Gyrate Atrophy, and central areolar choroidal dystrophy, which also shows choroid thinning and inflammation.^[^ ^15^, ^16^, ^17^ ^]^ Stargardt disease, a well‐studied genetically inherited maculopathy with mutations in the *ABCA4* gene, is characterized by reduced choroid thickness.^[^ ^18^ ^]^ Geographic atrophy, a type of late nonneovascular age‐related macular degenration (dry AMD), is characterized by the atrophy of the outer retina, RPE, and choriocapillaris, and there was no treatment until Pegcetacoplan (Syfovre) was approved by the FDA in 2023 which inhibits the complement system to reduce inflammation.^[^ ^19^, ^20^ ^]^ Choroidal ischemia might induce or aggravate AMD. The number of AMD patients globally was projected to be 196 million in 2020 and 288 million in 2040.^[^ ^21^ ^]^ Choroid thinning or atrophy is also frequently observed in pathological myopic patients, with about 40% of high myopic eyes showing myopic maculopathy.^[^ ^22^, ^23^, ^24^ ^]^ Therefore, choroidal atrophy is gaining attention in the studies of ocular degenerative diseases.^[^ ^25^, ^26^ ^]^

Human pluripotent stem cells (hPSC) can differentiate into a variety of cell types of the eye, including RPE cells.^[^ ^27^ ^]^ Clinical trials of hPSC‐RPE transplantation to treat AMD are ongoing around the globe and have shown to be safe and could stop the progression of AMD.^[^ ^28^, ^29^ ^]^ Since choroid pathology is common in retinopathies, we hypothesize that hPSC‐derived endothelial cells (ECs) may repair the choroid vasculature and rescue choroid atrophy, which would also be beneficial to protect against neurodegeneration in the eye.

Previously, we formulated a simplified, cost‐effective system to generate ECs from hPSCs.^[^ ^30^ ^]^ These ECs exhibited robust revascularization ability when transplanted to murine ischemic disease models, validating their therapeutic value in regenerative medicine. In this study, we established a rat model of choroidal ischemia (CI). We showed that transplantation of choroidal endothelial cells derived from human ESC and iPSC resulted in the formation of choroidal blood vessels, effectively reversing the pathological changes induced by ischemia. These findings support the use of hPSC‐derived ECs to treat ischemic diseases in the eye.

## 2 Results

### 2.1 Differentiation of Choroid Endothelial Cells from Human Pluripotent Stem Cells

**Figure 1:**
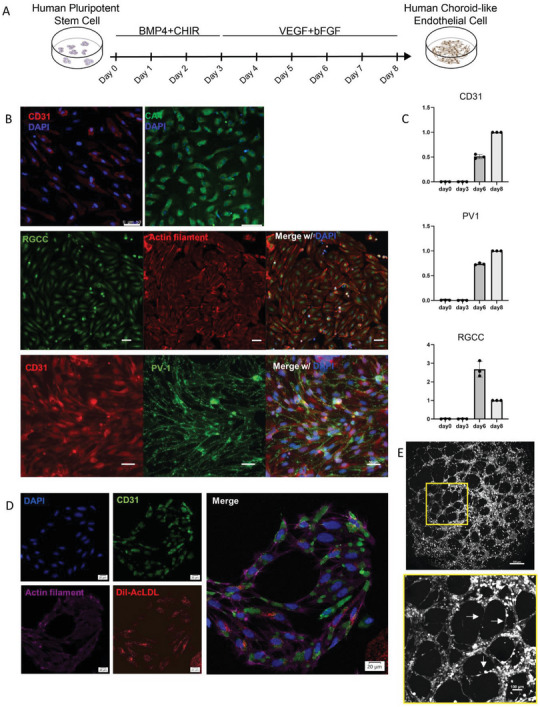
Differentiation of CECs from hPSCs. A) Schematics of CECs differentiation from hPSCs. B) Immunofluorescence of Day 8 ECs stained for choroidal EC marker CD31, CA4, RGCC, and PV‐1, and actin filament. Scale bar = 50 µm. C) QPCR of the choroidal EC marker genes expression relative to GAPDH at Day 0, 3, 6, and 8 of differentiation, and the value at Day 8 is set to “1”. (*n* = 3 biological repeats; error bars, means ± SEM.) D) Confocal images of Dil‐AcLDL ingestion assay. hPSC‐ECs labeled with CD31 (green) ingested Dil‐AcLDL (red), DAPI (blue), and actin filament (violet). Scale bar = 20 µm. E) Capillary‐like tubes formed(arrows) by hPSC‐ECs when plated on Matrigel for 8 h. Scale bar = 500 µm. Yellow box scale bar = 100 µm.

To obtain endothelial cells (ECs) with choroid endothelial characteristics from human pluripotent stem cells (hPSC), we used a two‐stage, monolayer, and chemically defined system to differentiate hPSC into ECs (**Figure** **1**A). This method was previously shown to induce nearly 100% *MESP1+* mesodermal progenitor cells at the end of stage one and have a high yield of EC at the end of stage two (Figure S1A, Supporting Information).^[^ ^30^, ^31^ ^]^ A lineage tracing study in mouse embryos showed that choroidal endothelial cells (CEC) were descendants of *MESP1+* mesoderm progenitor cells (Figure S1C, Supporting Information). We analyzed CEC marker expression in our hPSC‐ECs. Immunostaining study showed that endothelial marker CD31,^[^ ^32^ ^]^ capillary marker RGCC,^[^ ^33^ ^]^ choriocapillaris marker CA4,^[^ ^34^ ^]^ and fenestrae‐associated protein PV‐1^[^ ^35^ ^]^ were uniformly expressed in day 8 ECs (Figure 1B). QPCR confirmed that the expression levels of the choroidal endothelial cell markers CD31, PV1, and RGCC increased significantly on day 6 and day 8 of differentiation (Figure 1C). Dil‐Ac‐LDL uptake assay and tube formation assay showed that ECs possessed typical EC physiological functions including metabolism and capillary‐forming on day 8 (Figure 1D,E). SEM images of the in vitro differentiated EC showed some pore‐like structures mimicking the fenestration found in vivo (Figure 1B, Supporting Information). The diameters of these fenestrations range from 100 to 200 nm, consistent with the findings of prior studies.^[^ ^36^, ^37^, ^38^ ^]^ These results indicate that our hPSC‐EC resembles native CEC and can be used in transplantation studies.

### 2.2 A Rat Model of Choroidal Ischemia Resembled Pathological Changes in the Human Choroid

**Figure 2:**
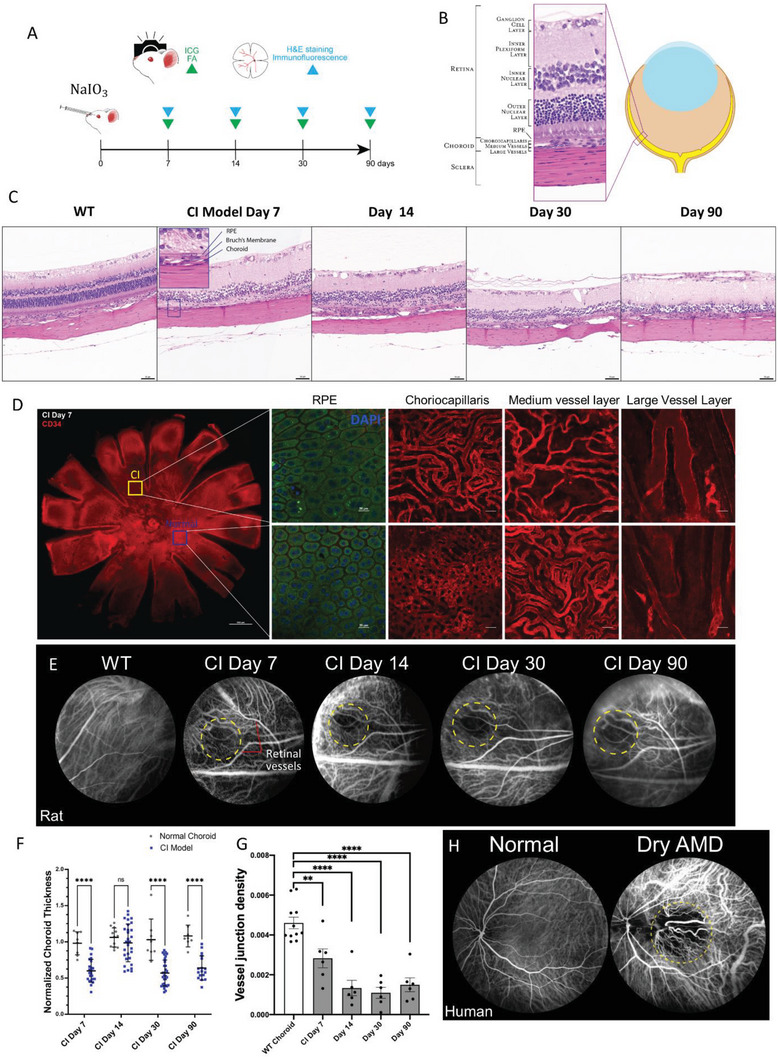
Establishment of a rat choroidal ischemia model. A) Schematic representation of the rat choroidal ischemia model. (FA, ICGA = Fluorescein Angiography and Indocyanine Green Angiography). B) Histological representation of the retina, choroid, and sclera. C) Representative H&E staining of wild‐type (WT) and sodium‐iodate (NaIO_3_) treated region in Choroidal Ischemia (CI) model eyes at different time points. Choroid thinning and outer retinal outer layer disruption in the CI model. Scale bar = 50 µm. D) Immunofluorescence of normal and CI choroid flatmounts at different levels (RPE, choriocapillaris, medium vessel layer, and large vessel layer, as indicated). CD34 (red), DAPI (blue), RPE autofluorescence (green). Choriocapillaris and medium vessel layers' atrophy, and RPE cell expansion in CI model. Scale bar = 20 µm. E) Representative FA/ICGA images of normal and CI regions. Vessel density decreased in CI mode. Local choroid atrophy in the CI model (yellow circle) was similar to that of the dry AMD patient's fundus (Figure 2H). F) Quantification of the normalized choroid thickness (NCT) from normal and CI eyes. (*n* = 119, biological replicate = 6, *p* values were obtained by unpaired *t*‐test). G) Quantitative analysis of the average vessel junction density in a normal eye and region (*n* = 11 for normal eye, *n* = 6 for CI, *p* values were obtained by comparing with WT choroid by multiple unpaired *t*‐tests). H) FA/ICG images of a normal human eye and an eye diagnosed with dry AMD. ^∗^ *p* < 0.05, ^∗∗^ *p* < 0.005, ^∗∗∗^ *p* < 0.0005, ^∗∗∗∗^ *p* < 0.00005. Error bar = Mean ± SD.

To establish a choroid blood vessel atrophic animal model to recapitulate the choroidopathologies, we injected a potent oxidant, NaIO_3_ (previously shown to induce retinal cell death^[^ ^39^, ^40^, ^41^ ^]^)_,_ into the suprachoroidal space to induce choroid atrophy. To confirm the presence of choroid atrophy, we conducted H&E staining, immunostaining, fluorescein angiography, and indocyanine green angiography (FA/ICGA) examinations at various time points, as illustrated in **Figure** **2**A. H&E staining showed tissue structure damage from the choroid to the outer nuclear layer 7 d after the NaIO_3_ injection. The lesion was present for at least 90 d (Figure 2C). The inner nuclear layer, the inner plexiform layer, and the ganglion cell layer had various degrees of disruption, while the retinal blood vessels were largely intact (Figure 2C). The Bruch's membrane, which separates the RPE and the choroid, was visible on H&E stained sections. Therefore, the thickness of the choroid can be measured as the distance between Bruch's membrane and the sclera from H&E images. A normalized choroid membrane thickness (NCT) was calculated as the ratio of the choroid thickness at the CI region (three random sites) versus that from the unaffected region (the nasal side) (Figure S2, Supporting Information). For the wild‐type control, the NCT was the ratio of the choroid thickness at the temporal side versus that at the nasal side. Quantitative analysis indicated that the choroid thickness was reduced by half on days 7, 30, and 90 postinjection (Figure 2F). On day 14, no significant difference in choroid thickness was observed, possibly due to local edema (Figure 2F). To examine the damage to the choroid blood vessels, eyes from CI model rats were harvested, flat‐mounted, and stained with an antibody reacting with both human and rat CD34 (h&rCD34), a marker membrane protein for endothelial cells (Figure 2D). The RPE cells in the nonischemic region had a regular, hexagonal shape which could be recognized by their autofluorescence under the confocal microscope. But in the center of the CI region, the RPE became atrophic, probably due to the high concentration of NaIO_3_. In the outer rim of the CI region, the RPE showed signs of damage where they decreased in cell number and lost their hexagonal shape. At the same time, their size increased in compensation (Figure 2D). The choriocapillaris, the uppermost layer of the choroid, was a highly anastomosed dense meshwork of capillaries in the intact region. The CI lesion resulted in a sparser vessel structure (Figure 2D). The medium vessel layer was also damaged and atrophic (Figure 2D). The deep vessel layers from normal and CI regions did not show significant differences in terms of shape and robustness (Figure 2D). Thus, the immunofluorescence staining of the CI choroid flat mount suggested it was the choriocapillaris and the medium vessel layers that were damaged the most by NaIO_3_.

FA/ICGA is a common practice in ophthalmology to examine the condition of the retinal and choroid blood vessels. FA/ICGA images of the CI quadrant appeared as a nonvascularized dark area, substantiating the atrophy of the choriocapillaris (Figure 2E). No vessel leakage or atrophy was observed in the superficial layer of retinal vessels, which generally appeared thicker (Figure 2E), indicating that the suprachoroidal injection damage was limited only to the choroid vessels. Quantitative analysis of blood vessel junctions per unit area using AngioTool (Figure S3, Supporting Information) showed a significant reduction from 7 d postinjection and lasted for 90 d, which was the end of the study (Figure 2G). To determine the relevance of our CI rat model, we obtained FA/ICGA images from a healthy patient and a patient diagnosed with typical dry AMD (Figure 2H). The choroid region of dry AMD patients has a dark area similar to that of the CI rat models, indicating that choroidal atrophy was successfully reproduced in our rat CI model.

### 2.3 hPSC‐ECs Expressed Angiogenesis, Immune‐Modulatory, and Neuroprotective Genes on Ex Vivo Ischemic Choroid

**Figure 3:**
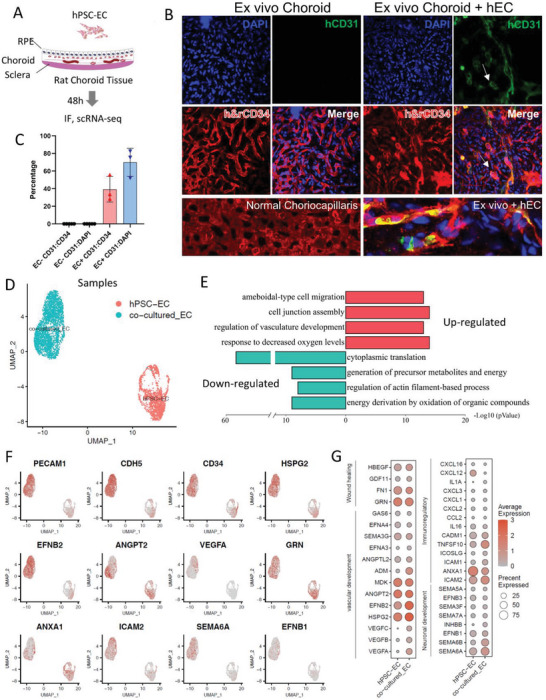
hPSC‐EC integrated into rat choroid in vitro. A) Schematics of the coculture experiment of isolated rat choroid and hPSC‐EC. B) Immunofluorescence of rat choroid and choroid cocultured with hPSC‐EC for 48 h. r&hCD34 (red), hCD31 (green), DAPI (blue); (*n* = 4, Scale bar = 20 µm.). Bottom: in vivo choriocapillaris and the enlarged section of hEC on ex vivo rat choroid. C) Quantitative analysis of the percentage of hCD31‐positive cells relative to CD34+ or DAPI+ cells in coculture images. Three representative images from three biological replicates were analyzed for each group. D) UMAP plot of human endothelial cells before and after coculture. E) Enriched GO terms in top 500 up and down‐regulated genes in ECs after coculture. F) UMAP plot showing normalized expression of endothelial marker genes and ligand genes. G) Bubble plot showing average expression of selective ligand genes in ECs before and after coculture.

To examine how hPSC‐ECs react to the ischemic choroid, we dissected the rat choroid/sclera complex and added human ECs to coculture with them (**Figure** **3**A). After 48 h of coculture, the choroid alone and choroid plus hPSC‐EC coculture were extensively washed, fixed, and stained with h&rCD34, human‐specific hCD31, and human nuclei antibodies (Figure 3B; Figure S4C, Supporting Information). The cultured choroid exhibited ischemia‐like morphology where the dense meshwork of capillaries appeared deflated and diminished. Moreover, the h&rCD34 staining reduced significantly compared to in vivo choriocapillaris (Figure 3B, left bottom panel). Such phenotype suggests that losing the blood supply has induced choroid atrophy. Interestingly, cocultured hPSC‐ECs highly expressed CD31 and formed vessel‐like structures marked by the staining of hCD31 and DAPI (Figure 3B ex vivo Choroid + hEC; Video S1, Supporting Information). Quantitative analysis of immunostaining images confirmed that in the coculture group, about 70% of total cells (DAPI+) were hCD31+. Among all the h&rCD34+ cells, 40% were hCD31+. There were no hCD31+ hPSC‐ECs in the rat Choroid alone group (Figure 3C). We next performed single‐cell RNA‐seq (scRNA‐seq) to compare hPSC‐EC before and after coculture with ex vivo ischemic rat choroid. Interestingly, the cocultured hPSC‐EC population separated from differentiated ECs (Figure 3D). Gene ontology significantly upregulated after coculture includes a response to decreased oxygen levels, regulation of vasculature development, cell junction assembly, and cell migration (Figure 3E), suggesting that endothelial cells transplanted in the injured eye area could regulate angiogenesis and possibly facilitate its recovery. In contrast, down‐regulated genes are involved in translation, regulation of actin filament‐based process, and energy derivation by oxidation (Figure 3E). hPSC‐ECs appeared to enhance their endothelial fate after interaction with the ex vivo ischemic choroid as they significantly upregulated *PECAM1*/*CD31*, *CD34*, and *CDH5* (Figure 3F; Figure S4A, Supporting Information). Moreover, there are very few non‐EC human cells in the hPSC‐EC group after 48 h coculture (Figure S4B, Supporting Information), confirming the purity of our EC preparation.

We then analyzed the secreted cytokines and extracellular proteins in hPSC‐ECs and discovered that many genes related to endothelial cell migration, proliferation, and vascular development were enriched (Figure 3F,G). Angiogenesis factors *VEGFA*, *HSPG2*, and *EFNB2* were upregulated after coculture. The vascular extracellular matrix gene *HSPG2/Perlecan* has been reported to promote the reconstitution of the blood‐brain barrier damaged by ischemic stroke by interacting with VEGF‐VEGFR2, FGF2, and PDGF to promote angiogenesis and wound healing.^[^ ^42^ ^]^ *EFNB2* encodes the ligand of receptor tyrosine kinase EphB4 and plays a critical role in vasculature development, sprouting angiogenesis, and vascular morphogenesis.^[^ ^43^ ^]^ EphrinB2‐Fc has been developed as a drug to protect vascular endothelium and enhance angiogenesis in patients with Kawasaki disease.^[^ ^44^ ^]^ HPSC‐ECs also upregulated genes such as *ANXA1, ICAM2, ICAM1, and ICOSLG*, which are involved in immune cell migration and recruitment, and cytokine and chemokine genes, including *IL1A*, *IL16*, *CCL2*, and *CXCL1* which involved in immunoregulatory and inflammatory processes, suggesting that they may modulate inflammation and immune response in diseased areas.^[^ ^45^, ^46^, ^47^, ^48^, ^49^ ^]^ After 48 h coculture, hPSC‐ECs also expressed *SEMA6A*, *EFNB1*, *SEMA7A*, *SEMA3F*, *EFNB3*, and *SEMA5A* at low levels, which have supportive roles in neuronal development.^[^ ^50^, ^51^, ^52^, ^53^, ^54^ ^]^ For example, *SEMA6A* has a critical role in regulating the development of retinal circuits.^[^ ^51^ ^]^ *SEMA5A* was implicated in retinal neural circuit formation.^[^ ^53^ ^]^ *SEMA3F* was reported to protect against subretinal neovascularization and is considered a promising target for treating pathological neovascularization formation in AMD.^[^ ^54^ ^]^ *GRN/Progranulin* is a secreted, immune regulatory protein and a new therapeutic target for neurodegenerative diseases.^[^ ^55^ ^]^ It was reported that during abnormal choroidal neovascularization (CNV), lower GRN expression resulted in elevated production of pro‐angiogenic VEGF‐A and pro‐inflammatory cytokines, leading to a larger CNV area.^[^ ^56^ ^]^ Thus, higher GRN levels in hPSC‐ECs after coculture may help to reduce excessive inflammation and neovascularization.

These findings imply that hPSC‐ECs could have many beneficial influences to improve the complex microenvironment in eye disease related to CI.

In contrast to in vivo choroid, choroidal ECs (rCECs) on in vitro cultured choroid lost EC identity, as there is significant downregulation of EC marker genes *Vwf*, *Cd34*, and *Cdh5* (Figure S5, Supporting Information). This result agrees with the immunostaining pattern, where CD34 intensity on in vitro cultured choroid is significantly weaker than that on in vivo choriocapillaris (Figure 3B, left bottom panel). It also suggests that rat choroidal ECs may undergo an endothelial‐to‐mesenchymal transition (EndMT) when there is no blood supply.

Together, these results demonstrated that our differentiated ECs responded to the in vitro ischemic choroid, strengthened their endothelial fate, and elevated many angiogenesis, immune‐modulatory, and neural protective genes, which potentially could reprogram the CI microenvironment.

### 2.4 hPSC‐ECs Integrated into Choroidal Vasculature

**Figure 4:**
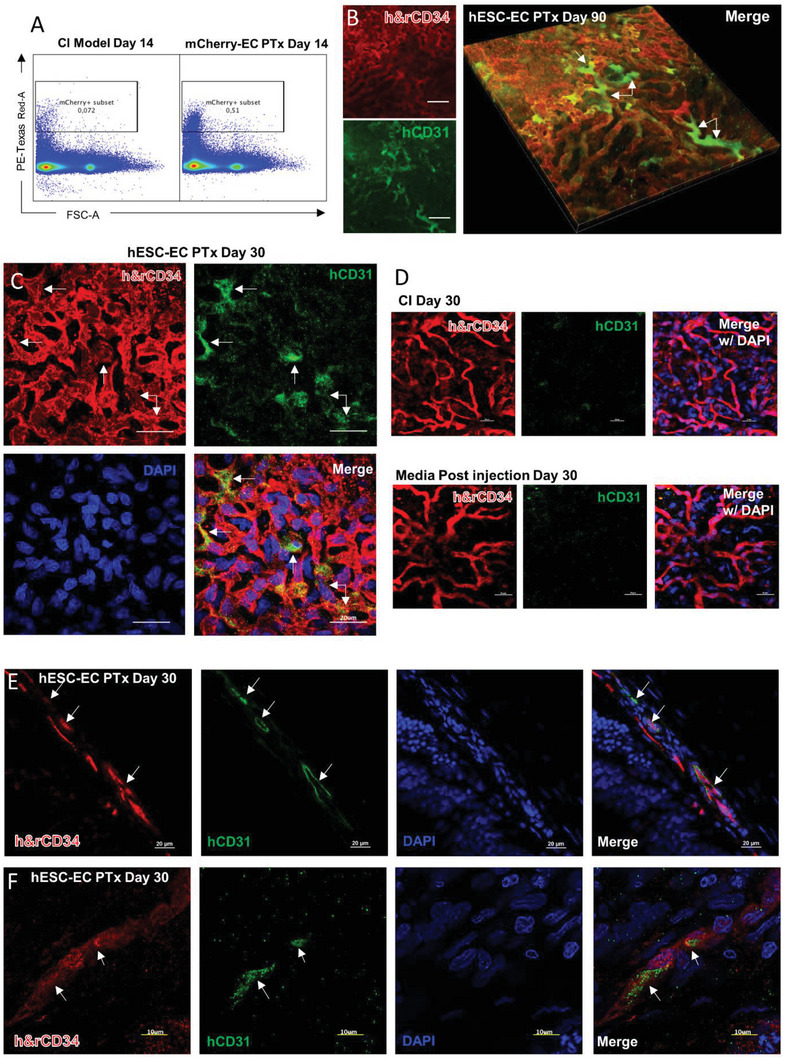
hPSC‐EC integrated with the rat choroid vasculatures after transplantation into the CI model. A) Flow cytometry analysis of the CI model choroid and CI model choroid transplanted with mCherry‐EC. Note that RPE cells can be detected in the PE‐Texas Red‐A channel due to their strong autofluorescence. In the mCherry ECs transplanted group, significant more mCherry+ cells were in the PE‐Texas Red‐A channel. (*n* = 3 independent experiments). B) Representative IF images of r&hCD34 (red), hCD31(green), and 3D reconstruction of the choroid flatmount 90 d after hPSC‐EC transplantation. Scale bar = 20 µm. C) Representative IF flatmount images from a rat CI model received EC transplantation 30 d post the transplantation. Scale bar = 20 µm. D) Representative IF flatmount images from a rat CI model and culture media injection 30 d post injection. Scale bar = 20 µm. E) Representative IF cryosection images from a rat CI model received EC transplantation 30 d post injection. Scale bar = 20 µm. F) Representative high magnification IF flatmount images from a rat CI model received EC transplantation 30 d post injection. Scale bar = 10 µm.

**Figure 5:**
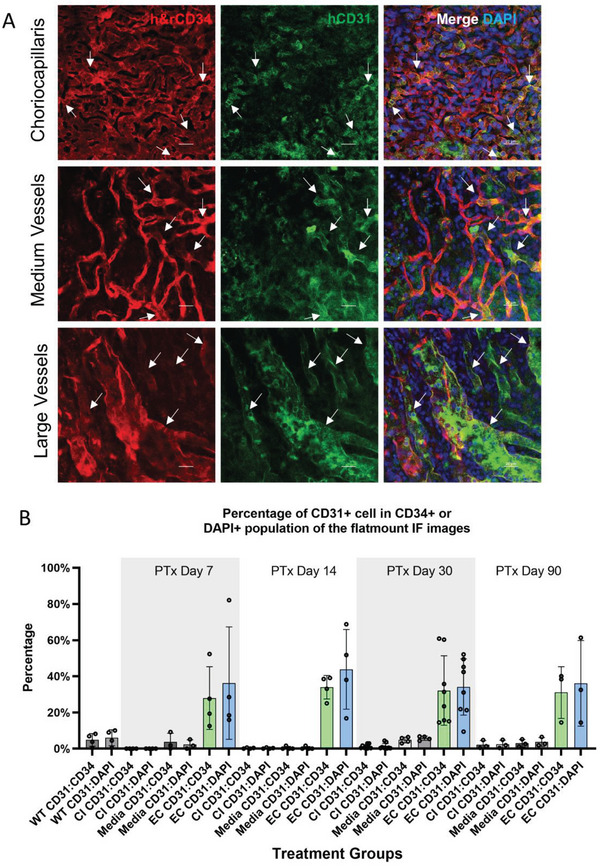
hPSC‐ECs integrated into all layers of rat choroid and formed chimeric vessels. A) Representative immunofluorescence (IF) images of three choroid layers in CI model stained with r&hCD34 (red), hCD31(green) Ab 7 d posttransplantation. Scale bar = 20 µm. B) Quantitative analysis of the percentage of hCD31‐positive cells relative to CD34+ or DAPI+ cells in EC transplantation images. Three representative images from three biological replicates were analyzed for each group.

Our transplantation experiments included three treatment groups: hPSC‐EC injected, noninjected, and EC culture medium injected. As the CI model showed significantly reduced vessel density as early as 7 d post‐NaIO_3_ injection (Figure 2G), we performed transplantation into the suprachoroidal space at this time point. The suprachoroidal injection of hPSC‐EC is illustrated in Figure S6 (Supporting Information) and various analyses were performed 7, 14, 30, 60, and 90 d after transplantation. To identify the grafted human ECs in CI eyes, we first transplanted H2B‐mCherry ECs. The 14 d after transplantation, eyeballs were dissected. The anterior chamber and the lens were first removed. The choroid and the retina were separated from the sclera, washed, and subjected to a range of assays. Flow cytometry analysis showed that in hPSC‐EC transplanted eyes, mCherry+ cells could be separated into a small but discernable population, making up around 0.51% of the total dissociated cells (**Figure** **4**A). In the non‐injected group, there were essentially no mCherry+ cells. These results proved that the transplanted hPSC‐ECs resided on the choroid and were viable 14 d after transplantation. For our immunostaining studies, we used a human‐specific hCD31 antibody to stain human ECs and h&rCD34, which reacts to both human and rat CD34, to label all ECs. Strikingly, ECs positive for both hCD31 and h&rCD34 could be seen as late as Day 90 (Figure 4B). In higher magnification images, we found tube‐like structures labeled by both hCD31 (green) and h&rCD34 (red) 30 d after transplantation, indicating hPSC‐EC integrated with rat choroid vessel networks (Figure 4C,E,F), while noninjected or medium injected CI choroid had no hCD31 signal (Figure 4D). HPSC‐ECs with strong hCD31 expression could be found on choriocapillaris, medium, and large vessel layers of the choroid (**Figure** **5**A). High magnification images identified hCD31+ h&rCD34+ small vessel cavities and walls 7, 30, and 60 d after transplantation (Figure S7A–C, Supporting Information), confirming that they have formed chimeric vessels with rat ECs. Quantification of hCD31+ cells from confocal images suggested that about 30–40% of cells in the view field were labeled with hCD31+ (Figure 5B).

**Figure 6:**
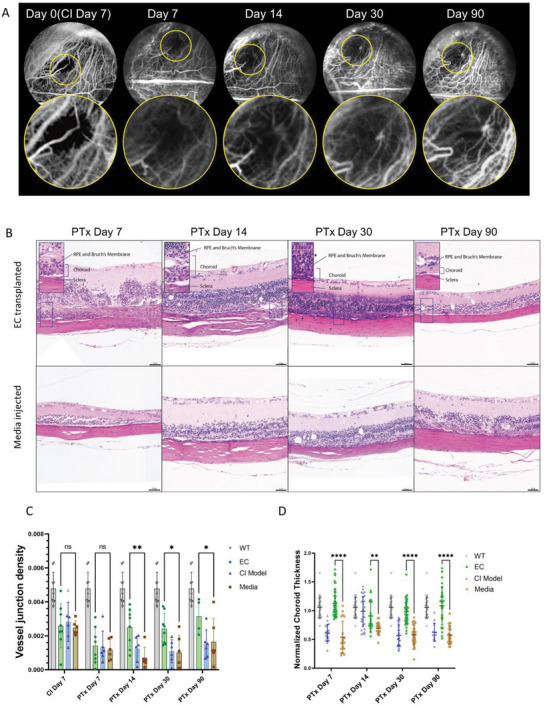
Choroid Thickness and FA/ICGA evaluations of the EC transplanted rat eyes. A) Representative FA/ICGA images from a rat CI model received EC transplantation 7, 14, 30, and 90 d post the procedure. The yellow circle highlighted newly formed vessels. B) Representative H&E staining of EC‐transplanted and media‐injected eyes at different time points. Enlarged regions showing choroid became thicker after EC transplantation. Scale bar = 50 µm. C) Quantitative analysis of the average vessel junction density based on FA/ICGA images from normal and EC‐injected CI rats. Statistical significance is calculated between the EC transplanted group and the cell media injected group and represented by asterisks. (*n* = 6, *p* values were obtained by unpaired *t*‐test between EC and media group). D) Quantification of the normalized choroid thickness based on H&E staining images, Statistical significance is calculated between EC transplanted group and media injected group and is represented by asterisks. (*n* = 227, biological replicate = 6 rats per group, *p* values were obtained by unpaired *t*‐test between EC and media group). ^∗^ *p* < 0.05, ^∗∗^ *p* < 0.005, ^∗∗∗^ *p* < 0.0005, ^∗∗∗∗^ *p* < 0.00005. Error bar = Mean ± SD.

We performed an FA/ICGA examination of eyes injected with or without hPSC‐EC. In EC‐engrafted eyes, the ischemic area showed signs of newly developed blood vessels in the choroid layer, and superficial retinal vessels remained intact (**Figure** **6**A). Importantly, no vessel leakage or exudative choroid neovascularization was found in any FA/ICGA images of EC‐transplanted eyes. Newly developed blood vessels were seen as early as Day 7 and gradually increased in density and length until Day 90 (Figure 6A). Quantitative analysis of the extracted FA/ICGA image using AngioTool^[^ ^57^ ^]^ showed that EC injected group significantly increased blood vessel junction density compared to non‐injected and medium‐injected groups 14 to 90 d post‐transplantation (Figure 6C). There were no newly developed blood vessels in the culture medium injected group (Figure S8B, Supporting Information). H&E staining of the harvested eyes confirmed the thickening of the choroid after hPSC‐EC transplantation. To ensure the injection destination is not a subretinal cavity, we make sure the needle did not penetrate through the choroid and the Bruch's membrane into the subretinal cavity (Figure S6, Supporting Information). No bleeding was seen during the suprachoroidal injection procedure and the Bruch's membrane was intact in our histological section images (Figure 6B). We measured the normalized choroid thickness (NCT) in damaged sites 7, 14, 30, and 90 d after the CI procedure. The NCT of EC transplanted eyes reached the normal level on Day 30. On the contrary, noninjected and medium‐injected CI choroids were significantly thinner (Figure 6D), indicating fewer blood vessels in the choroid.

**Figure 7:**
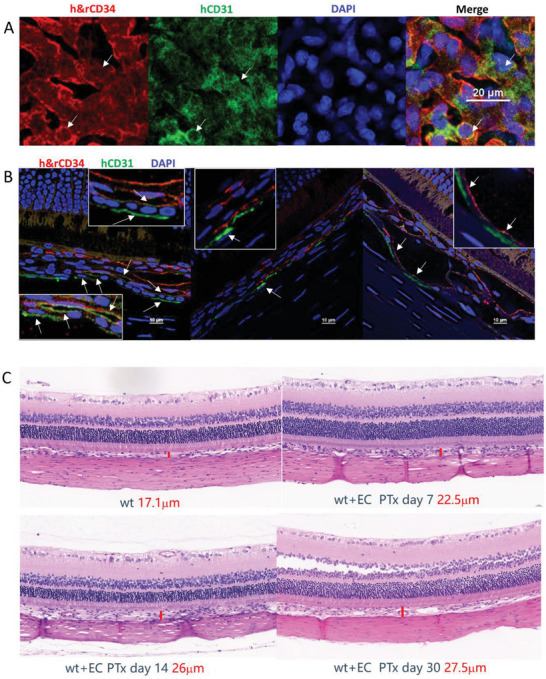
hPSC‐ECs can integrate into undamaged rat choroid blood vessels. A) Representative flatmount IF images of H1‐ECs integrated with undamaged choroid blood vessels. Scale bar = 20 µm. B) Representative IF cryosection images of the undamaged choroid with H1‐ECs. Scale bar = 10 µm. C) Representative H&E staining of EC‐transplanted eyes at undamaged regions during different time points. Scale bar = 20 µm.

We also observed hPSC‐ECs integrated with the rat choroid blood vessels in areas next to the damaged sites. As shown in **Figure** **7**, they readily integrated with the rat choriocapillaris judging by the extensive colocalization of hCD31 and h&rCD34 signals (Figure 7A). Staining on cross sections revealed hCD31 lining the choroidal vessel walls (Figure 7B), indicating the formation of human or chimeric blood vessels. The NCT of undamaged choroid injected with hPSC‐EC also increased (Figure 7C).

Mesenchymal stem cell (MSC) transplantation has been used to treat eye diseases in animal models and human patients.^[^ ^58^ ^]^ To compare with hPSC‐EC transplantation, we transplanted human umbilical cord‐MSC (UC‐MSC) into our CI model. However, severe intraocular inflammation occurred in the UC‐MSC transplanted eyes. Consequently, FA/ICGA images appeared fuzzy. The structure of the choroid was disrupted and shunting vessels could be found (Figure S8C–E, Supporting Information). H&E staining showed synechia of the iris and inflammatory cell infiltration on the surface of the retina, vitreous cavity, and anterior chamber (Figure S8E, Supporting Information). On the other hand, intraocular inflammation was never detected in the hPSC‐ECs group (Figure S8A, Supporting Information). The above results demonstrated that hPSC‐ECs were lowly immunogenic and could form and regenerate choroid blood vessels in the CI model.

### 2.5 HPSC‐EC Transplantation Increased Retinal Activity

Last, we investigated the effects of EC transplantation on retinal functions 30 d post‐transplantation using electroretinogram (ERG) assessments. ERG graphs from wildtype (WT) and CI model rats revealed that the retinal activities were significantly reduced after NaIO_3_ injection.

**Figure 8:**
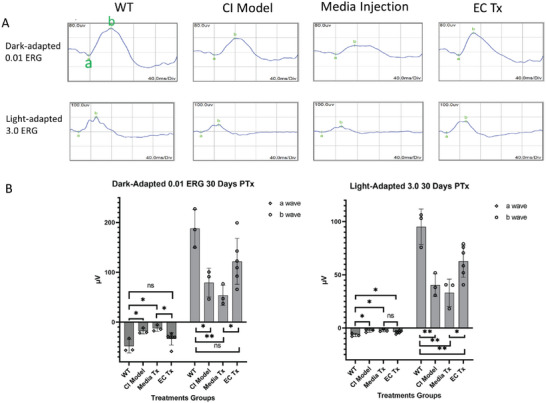
Electroretinogram (ERG) of rats 30 d posttreatments. A) Waveforms of ERGs in different groups. Dark‐adapted 0.01 ERG represents the function of rod cells (a‐wave) and their downstream bipolar cells (b‐wave). Light‐adapted 3.0 ERG represents the function of cone cells (a‐wave) and their related bipolar cells (b‐wave). The amplitude of a‐waves and b‐waves decreased after NaIO_3_ injection in the CI model, and not increased after media injection, while improvement was observed after EC transplantation. B) Quantifications of a‐waves and b‐waves. a‐waves and b‐waves in the EC transplantation group improved significantly than that of the medium injection group. (WT: *n* = 4, CI only: *n* = 4, Media Tx: *n* = 4, EC Tx:*n* = 6, unpaired *t*‐test, *p < 0.05, **p < 0.005, Error bar = Mean ± SD).

The amplitude of a‐waves and b‐waves decreased in the CI model. However, after transplantation of EC to the choroid, we observed considerable improvements in dark‐adapted 0.01 ERG responses, which presented the function of rod cells (a‐wave) and its downstream bipolar cells (b‐wave) (**Figure** **8**A,B). As rodents' photoreceptors are predominantly composed of rods, the observed enhancement in dark‐adapted ERG signifies a beneficial outcome following hPSC‐ECs transplantation. These findings suggest a potential therapeutic approach for addressing retinal dysfunction associated with choroidal ischemia through repairing endothelial cells, offering promising prospects for future clinical applications in vision restoration.

## 3 Discussion

In this study, we showed that hPSC‐ECs have CEC characteristics. Upon transplantation into the suprachoroidal space of the rat CI model, hPSC‐ECs were integrated into choriocapillaris, medium, and large choroid vessels for up to 90 d. They also rescued the atrophic changes in the CI without detectable adverse effects. Therefore, hPSC‐EC transplantation might be a promising strategy for treating CI.

Choroid thinning or degeneration prevents the adequate exchange of biological molecules and contributes to the deterioration of AMD and myopia.^[^ ^5^, ^59^ ^]^ Dry AMD, which accounts for 80% of AMD, has a strong correlation with CI.^[^ ^60^ ^]^ In the wet form of AMD, choriocapillaris atrophy occurs before RPE degeneration, indicating that neovascularization might also be a maladaptive response to ischemia.^[^ ^8^ ^]^ In our CI model, the transient presence of NaIO_3_ in the suprachoroidal space caused localized but severe damage to the choroidal blood vessels. Compared to laser‐induced RPE ablation and CNV model,^[^ ^25^ ^]^ our procedure induced primary choroidal blood vessel damage and CI. Imaging and histological analysis of the CI model showed atrophic choroidal vasculature and disruption in adjacent RPE and photoreceptor layers but no hemorrhage, neovascularization, or retinal detachment. The atrophic area is limited to the temporal quadrant of the choroid. Thus, the unaffected regions can serve as controls. Similar to patients diagnosed with dry AMD, the FA/ICGA images from our rat CI model have darkened areas, indicating atrophy of choriocapillaris (Figure 2E). These results suggest that our CI model could be clinically relevant choroid atrophy disease model. In our CI model, as the damage was primarily to the choroid, we injected hPSC‐ECs into the suprachoroidal space, which differs from the intravitreal injection. Remarkably, hPSC‐ECs formed chimeric vessels at choriocapillaris, medium, and large choroid vessels for up to 90 d and increased the thickness of the choroid in ischemic areas (Figures 4, 5, and 6). Moreover, hPSC‐ECs could also integrate with choroidal vasculature at undamaged regions, suggesting they have the ability to migrate within the choroid. Impressively, EC transplantation improved the visual function of CI rats (Figure 8). These findings have significant clinical implications. As there is still no effective treatment for choroidal ischemia (CI), hPSC‐EC transplantation could potentially restore choroid blood vessels, improve the microenvironment, and protect the RPE and photoreceptors. Our results will also serve as an important source of information for other studies in this area.

In human clinical trials, hPSC‐derived endothelial cells or endothelial progenitor cells have been used in cell therapy to treat ischemic and idiopathic artery diseases (NCT03098771, NCT00372346, NCT01468064). Recently, Gil and colleagues reported that hiPSC‐derived KDR+CD56+APLNR+ cells can integrate into the retinal vessels of diabetic mice.^[^ ^61^ ^]^ The KDR+CD56+APLNR+ cells are similar to the MESP1+ cells (CD13+CD56+) described in our previous study.^[^ ^31^ ^]^ In this study, we used purified ECs differentiated from MESP1+ cells. Several features of our ECs make them the suitable cell type for transplantation therapy to treat CI. First, they can be produced for large amounts in high purity cost‐effectively using Xeno‐free and chemically defined simple medium.^[^ ^30^ ^]^ Second, many genes related to immune recognition, such as *HLA‐A/B/C*, *PTGS1/2*, *PTGES2/3*, *NOS3*, *IL32*, *CIITA*, and *B2M*, were significantly downregulated in ECs differentiated in AATS medium compared to primary HUVECs and HDMECs.^[^ ^30^ ^]^ Therefore, these ECs will exhibit low immunogenicity after transplantation into the suprachoroidal space. Third, in CI, the lost cells appeared to be the choriocapillaris and medium‐layer ECs. We showed that our hPSC‐ECs expressed capillary marker RGCC, choriocapillaris marker CA4, and PV‐1 (Figure 1), similar to CECs described in other studies.^[^ ^62^, ^63^, ^64^ ^]^ Importantly, they could incorporate with choriocapillaris, medium, and large vessels and lasted up to 90 d after transplantation (Figure 4). A recent clinical trial reported survival of allogenic hESC‐RPE cells in the patient for 2 years without long‐term immunosuppression.^[^ ^65^ ^]^ We speculate that our hPSC‐ECs may also be less likely to be rejected in the choroid due to the low immunogenicity gene expression. hPSC‐EC transplantation to the CI model demonstrated a good safety profile. The regenerated choroidal blood vessels appeared normal in FA/ICGA imaging and confocal microscopy, and choroid thickness was restored. There was no overgrowth of hPSC‐ECs, nor inflammation or hemorrhage. On the contrary, we observed severe inflammation and proliferative vitreoretinopathy in eyes injected with the same number of UC‐MSCs (Figure S8, Supporting Information). This result agrees with the concern raised by Kuriyan et al.^[^ ^66^ ^]^ about untested autologous stem cell therapy for AMD eyes. For our hPSC‐EC transplantation study, 5 × 10^4^ or even a lower number of ECs was sufficient to show a reparative effect on rat CI. Compared to a nice study using anti‐VEGF antibodies conjugated to exosomes to inhibit CNV,^[^ ^67^ ^]^ culture supernatant from 1 × 10^7^ cells or more is needed for exosome preparation. Thus, cell preparation for EC transplantation to treat CI is much less labor‐demanding.

Moreover, the scRNA‐seq analysis revealed that when cocultured with ischemic choroid in vitro, hPSC‐ECs upregulate genes of many secretory proteins, including VEGFA, HSPG2, EFNB2, SEMA6A, SEMA5A, SEMA3F, GRN/Progranulin et al., that modulate endothelial cell migration, immune responses, retinal development, and inhibit CNV (Figure 3E–G). Interestingly, SEMA3F and GRN/Progranulin are new therapeutic targets for neurodegenerative diseases and CNV. Therefore, hPSC‐EC‐sourced SEMA3F and GRN/Progranulin may help to protect photoreceptor cells and reduce CNV. Thus, hPSC‐EC injection to CI models appeared to restore choroid blood vessels and improve the microenvironment, which would benefit the RPE and photoreceptors.

The hPSC‐RPE cells could survive long‐term after transplantation into human subjects.^[^ ^65^, ^68^ ^]^ Despite the success, RPE cells alone cannot rescue the loss of photoreceptors; therefore, visual improvement is not achieved.^[^ ^69^ ^]^ The health of RPE is reliant on choroid blood flow. The RPE will begin to deteriorate if the choroid blood vessels atrophy. Improving the retina microenvironment and circulation with hPSC‐EC may offer a strategy to boost retina regeneration. In the future, combinatory cell therapy using hPSC‐ECs together with RPE or photoreceptors may improve the efficacy of treating AMD.

In summary, our study showed promising results of hPSC‐ECs transplantation therapy for ischemic choroidopathy in a relevant animal model, which may inform a new treatment strategy for ocular diseases involving CI.

## 4 Limitations of the Study

Our study also has several limitations. It is unclear whether the regenerative effect was from transplanted hPSC‐ECs or the paracrine effect. Moreover, the change in cell states and tissue environment of the host choroid is unknown. Thus, additional analysis of the in vivo choroid tissue after cell transplantation using scRNA‐seq and proteomic approaches will inform the status of the choroid and retina and the repair mechanisms. In transplantation experiments, the volume of cells introduced into suprachoroidal space is very limited due to the intraocular pressure. We injected 5 × 10^4^ cells per rat eye, but only a small proportion of ECs remained. To improve the efficiency of EC retention, biomaterials as a scaffold for ECs may be helpful in future transplantation efforts. Finally, CNV is a potentially dangerous complication. However, CNV might result from local ischemia, excessive VEGF release, and subsequent uncontrolled vessel growth. We aim to repair choroid to improve retinal blood supply, which could lessen the likelihood of ischemia‐related CNV. In our study, terminally differentiated endothelial cells were transplanted, and no CNV was observed in any of the subjects until the study ended in 90 d. Therefore, long‐term observation will be required to completely rule out the CNV risk.

## 5 Experimental Section

### hPSC Culture and Differentiation

H1, H9 hESCs (WiCell Institute), MESP1‐mTomato reporter hESC line,^[^ ^31^ ^]^ H2B‐mCherry hESC line,^[^ ^70^ ^]^ and hiPSCs^[^ ^71^ ^]^ were cultured on Matrigel (BD Biosciences)‐coated plates (Corning) in TeSR‐E8 medium (STEMCELL Technologies). The chemically defined medium AATS for differentiation was described in the previous study.^[^ ^30^ ^]^ For mesoderm progenitor cell induction, 5 ng mL^−1^ BMP4 (Peprotech) and 2 µm mL^−1^ CHIR99021 (Tocris) were added at the desired concentration, depending on the cell line. For EC induction, 50 ng mL^−1^ VEGF‐A (SinoBiological) and 10 ng mL^−1^ bFGF (FGF2, SinoBiological) were added. ROCK inhibitor Y27632 (TOCRIS) (5 µm) was added during passaging for 24 h and removed with the medium change.

### RNA Isolation, Reverse Transcription, and Q‐PCR Analysis

Total RNA was isolated from undifferentiated hESCs and purified ECs were collected on days 0, 3, 6, and 8 using TRIzol according to the manufacturer's instructions. RNA (1 µg) of each sample was reverse‐transcribed with 5 × All‐In‐One RT MasterMix. Q‐PCR reactions were performed using GoTaq qPCR Master Mix (Promega) in a CFX96 Real‐Time System (Bio‐Rad), and results were analyzed with the Bio‐Rad CFX Manager program. The sample input was normalized against the Ct (Critical threshold) value of *GAPDH*. PCR primers were selected from PrimerBank^[^ ^72^ ^]^ and listed in the resource table.

### Flow Cytometry Analysis

Cells were dissociated into single‐cell suspension with accutase (Millipore) or rinsed off from the plate wherever suitable and resuspended in a FACS washing buffer (PBS with 5% fetal bovine serum (FBS) and 2.5 mm EDTA). The cell suspension was stained with CD31‐FITC (1:500, BD 555 445) for 15 min at room temperature. Then, cells were washed and suspended in 5% FBS in phosphate‐buffered saline (PBS) buffer. Data were collected with a FACS Caliber flow cytometer (BD).

### Immunostaining, Image Acquisition, and Analysis

Cells were fixed with 4% paraformaldehyde, permeabilized in 0.5% Triton X‐100 (Sigma), blocked in 10% normal goat serum (Origene), and then incubated with primary antibodies. Antibodies used in this study included human CD31 (1:500, Santa Cruz SC81158), CD34 (1:500, Abcam, ab81289), STEM101(1:100, TAKARA, Japan), CA4(1:500, R&D MAB21861), RGCC PV‐1 (1:50, Abcam ab81719), Phalloidin 647‐conjugated (1:200, Abcam ab176759), at 4 °C overnight then detected by DyLight 488‐ or 549‐conjugated secondary antibodies (EarthOx). Nuclei were stained with DAPI (Sigma) or mounted with VECTASHIELD mounting media with DAPI (H‐1200‐10, Vector Lab). Immunofluorescence microscopy images were acquired with a Nikon A1 HD25 confocal microscope (Nikon, Japan). The images were processed with Fiji imageJ.^[^ ^73^ ^]^ The process was described by Shihan et al.^[^ ^74^ ^]^ Briefly, Nikon raw images with nd2 format were imported into ImageJ with the Bio‐formats plugin, and then remove‐outlier was performed. Then, the measure function was called to generate the area positive signal of each channel.

### Scanning Electron Microscopy (SEM)

hPSC‐ECs on a 35 mm cell culture dish were fixed with 2% paraformaldehyde/2.5 glutaraldehyde in cacodylate buffer, dehydrated, dried in hexamethyldisalazane, gold‐coated, and examined under a JSM‐6700 field emission SEM (Zeiss Merlin).

### hPSC‐EC Functional Studies

The function of hPSC‐ECs was tested by DiI‐conjugated acetylated low‐density lipoprotein (DiI‐ac‐LDL) uptake and tube formation assays. For the DiI‐ac‐LDL uptake assay, hPSC‐ECs were incubated with 20 mg mL^−1^ of DiI‐Ac‐LDL (Yeasen) at 37 °C for 6 h, washed with PBS, and stained with Hoechst 33 258 (Dojindo). For the tube formation assay, 1 × 10^5^ cells were seeded in one well of 24‐well plates coated with Matrigel (BD) and incubated at 37 °C for 12 h.

### Animals

Specific pathogen‐free (SPF) grade Sprague Dawley male rats weighing 250 g were purchased from the Beijing Vital River Laboratory animal center. All animal experiments were approved by the Internal Review Board, Laboratory Animal Research Center, Tsinghua University. Protocol number 20‐NJ3.

### Rat Model of Choroidal Ischemia

NaIO_3_ (10 µg/1 µL) was injected into the suprachoroidal space of the rat's right eye. Briefly, the rat was first anesthetized by intraperitoneal injection of 2% pentobarbital with a dosage of 50 mg kg^−1^. Oxybuprocaine Hydrochloride Eye Drops were applied to the rat's right eye to induce topical ocular anesthesia. A 2 mm cut was made to the temporal conjunctiva to expose the sclera. A microliter syringe (Model 1701 RN SYR, Hamilton, NV, US) equipped with a 33G needle was used to access the suprachoroidal space. The needle was inserted with a minimal angle parallel to the optic nerve 2 mm lateral to the limbus. One microliter of NaIO_3_ solution was injected after the needle tip was 2 mm deep. Then, the rat eye was rinsed with PBS. The CI model was confirmed with Fluorescein Angiography (FA), Indocyanine Green Chorioangiography (ICGA), Hematoxylin and eosin stain (H&E Staining).

### Coculture of Rat Choroid and hPSC‐Derived Endothelial Cells

Rat choroid membranes were used for hPSC‐EC coculture. Dissected rat eyeballs were soaked in 70% ethanol for 10 s, rinsed with sterile PBS, then with DMEM/F12:EC differentiation medium (1:1). A circumferential cut along the cornea released the aqueous humour and the intraocular tension. After removing the iris, lens, and aqueous humour, four incisions were made to the cup‐shaped retina‐sclera complex, leaving a flower‐shaped flattened tissue. Under the microscope, the retina was carefully removed with a pair of straight‐notched tissue forceps (0.25 mm). The choroid‐sclera complex was cut into 2 mm squares with a pair of micro scissors and transferred to 24‐well tissue culture plates. For coculture, 0.2 million EC suspension was added to the choroid‐sclera complex in a 24‐well plate. The medium was changed daily. After 48 h of coculture, the choroid‐sclera complex was washed and fixed with 4% PFA.

### Single‐Cell RNA‐Sequencing

The choroid‐sclera complex as described in “Coculture of rat choroid and hPSC‐derived endothelial cells.” was prepared. The choroid‐RPE layer was then peeled off from the complex, followed by cell dissociation using Collagenase A (6.25 mg mL^−1^), Dispase II (6.25 mg mL^−1^), and DNase (62.5 µg mL^−1^; Roche) at 37 °C with continuous rotation for 15 min. Then 0.25% trypsin‐EDTA (Gibco) for 5 min at 37 °C was added to fully dissociate cells. The single‐cell suspension was filtered through a 70 µm cell strainer, centrifuged at 300 g for 3 min, and resuspended in DMEM + 5% FBS. Cells were then FACS sorted, and single‐cell RNA‐sequencing was performed following 10 × genomics’ instrument instruction.

### Single‐Cell RNA‐Sequencing Data Analysis

Raw data of the WT and coculture sample were mapped to a GRCh38 and Rnor_6.0 mixed reference genome using STARsolo pipeline (version:2.7.8a), which performed read mapping, cell demultiplexing, UMI counting, and initial cell filtering. ScRNA‐seq data are publicly available at the Gene Expression Omnibus (GEO) with accession number GSE235990. The generated UMI matrix was then analyzed using Suerat (V4). Cells with lower than 2000 UMIs and 1000 genes were filtered out, then cell clustering and UMAP embedding using the top 15 principal components were performed. The resolution in the FindClusters() function to 0.1 was set and cell types based on reported cell type markers were annotated. Endothelial cells in the coculture sample with a previously reported hiPSC‐derived EC dataset (GSE166462) were combined and differential expression analysis using the FindMarkers() function with the default Wilcoxon Rank Sum test was performed. To explore functional ligands secreted by ECs, all ligand genes in a curated ligand–receptor interaction database in CellChat(V1.5) package, manually selected genes based on their reported function in vascular development, immune modulation, wound healing, and nervous system development, were extracted and their expression using UMAP plot and dotplot was shown.

### Cell Transplantation to the Rat Model of Choroidal Ischemia

One microliter of 5 × 10^4^ hPSC‐EC, 5 × 10^4^ UC‐MSC, or Gibco RPMI 1640 culture medium (Thermo Fisher, USA) was injected in a similar manner as described in the CI model. A 2 mm cut was made to the temporal conjunctiva to expose the sclera. One microliter of cells or medium was injected with a microliter syringe (Model 1701 RN SYR, Hamilton, NV, US) equipped with a 33G needle into the suprachoroidal space. The needle was inserted with a minimal angle parallel to the optic nerve 2 mm lateral to the limbus, then hPSC‐EC was injected after the needle tip was 2 mm deep. To ensure the injection destination was not the subretinal cavity, it was made sure that the needle did not penetrate through the choroid and the Bruch's membrane into the subretinal cavity (Figure S6, Supporting Information). No bleeding was seen during the suprachoroidal injection procedure. After cell transplantation, immunosuppressant cyclosporine A was given orally at 250 mg kg^−1^ until sample collection.

### Fundus Fluorescein Angiography and Indocyanine Green Chorioangiography Analysis

Fundus fluorescein and indocyanine green angiography were carried out at 7, 14, 28 d, or 3 months under anesthesia (intraperitoneal injection of 50 mg kg^−1^ pentobarbital). The pupils were dilated with 0.5% tropicamide and 0.5% phenylephrine hydrochloride eye drops. Heidelberg retinal angiography system (HRA 2, Germany) was used to evaluate the retina and choroidal vessel changes 5 min after intraperitoneal injection of fluorescein sodium (60 mg kg^−1^; Akron, Buffalo Grove, IL) and indocyanine green dye (6 mg kg^−1^; Akron, Buffalo Grove, IL). The rat eyes were manually positioned in front of the angiography camera to get the optimal view. The operation and interpretation of angiography images were performed by two ophthalmologists. The images were analyzed with AngioTool.^[^ ^57^ ^]^

### Immunofluorescence on RPE/Choroidal Flatmounts and Histology Sections

Rats were euthanized at 7, 14, 30 d, or 3 months after injection of NaIO_3_. Eyes were enucleated and processed for RPE/choroidal flatmount immunofluorescence staining (*n* = 6 at each age). The anterior segments and retinas of the eyes were removed. The RPE/choroid complex was fixed in 4% paraformaldehyde (PFA) diluted in Phosphate Buffered Saline (PBS) at 4 °C overnight. The RPE/choroid flat mounts were blocked in 5% goat serum (prepared in PBS) for 4 h before incubation in primary antibodies overnight at 4 °C.

### Rat Electroretinogram (ERG)

All rats were dark adapted for 12 h before experiments and the experiments were carried out in red ambient light condition. In brief, the rats were anesthetized with 3 mL kg^−1^ 1% Pentobarbital. Corneal anesthesia was achieved with a single drop of proparacaine hydrochloride (0.5%, Alcon Laboratories Inc.). After pupil dilation with 0.5% tropicamide and 0.5% phenylephrine hydrochloride eye drops, the rats were secured and attached with electrodes. Flash electroretinogram (FERG) was conducted with electrodes position: cornea: +; cheek: ‐; tail subcutaneous: ground. Then the experiments were conducted according to OPTO‐III (Optoprobe Science Ltd. UK) user manual.

### Statistical Analysis

Quantitative data were represented as mean ± standard deviation (SD) as indicated in the figure legend. The statistical significance was determined by unpaired *t*‐test (two‐tail) for two groups using Prism Graphpad 9.5. *p* < 0.05 was considered statistically significant.

## Conflict of Interest

The authors declare no conflict of interest.

## Author Contributions

M.L., P.W., S.T.H., and H.Q. contributed equally to this work. M.L., P.W., S.H., J.N., and Y.H. conceived and designed the experiments. M.L., P.W., and S.H. performed cell differentiation, cell transplantation, rat CI model establishment, and phenotype analysis. H.Q., S.L., Y.J., Y.Z., J.L., and J.G. contributed to cell differentiation, characterization, and data assembly. M.L., P.W., S.H., J.N., and Y.H. wrote the manuscript. All authors approved the manuscript.

## Supporting information

Supporting Information

Supplemental Video 1

Supplemental Video 2

## Data Availability

The data that support the findings of this study are openly available in Gene Expression Omnibus at https://www.ncbi.nlm.nih.gov/geo/, reference number 235990.
