# Supplementary material for: Human Pluripotent Stem Cells Derived Endothelial Cells Repair Choroidal Ischemia
Source: Adv Sci (Weinh). 2023 Dec 19;11(9):2302940. doi: 10.1002/advs.202302940 (PMC10916649; doi:10.1002/advs.202302940)
Supplement: Supplementary file 1 — Supporting Information [file ADVS-11-2302940-s001.pdf]

ADVANCED SCIENCE
Open Access

Supporting Information

for *Adv. Sci.*, DOI 10.1002/advs.202302940

Human Pluripotent Stem Cells Derived Endothelial Cells Repair Choroidal Ischemia

Mengda Li, Peiliang Wang, Si Tong Huo, Hui Qiu, Chendi Li, Siyong Lin, Libin Guo, Yicong Ji, Yonglin Zhu, Jinyang Liu, Jianying Guo, Jie Na and Yuntao Hu**

Supplemental Figure 1. Choroid endothelial cells differentiated from Mesp1 progenitor stem cells.

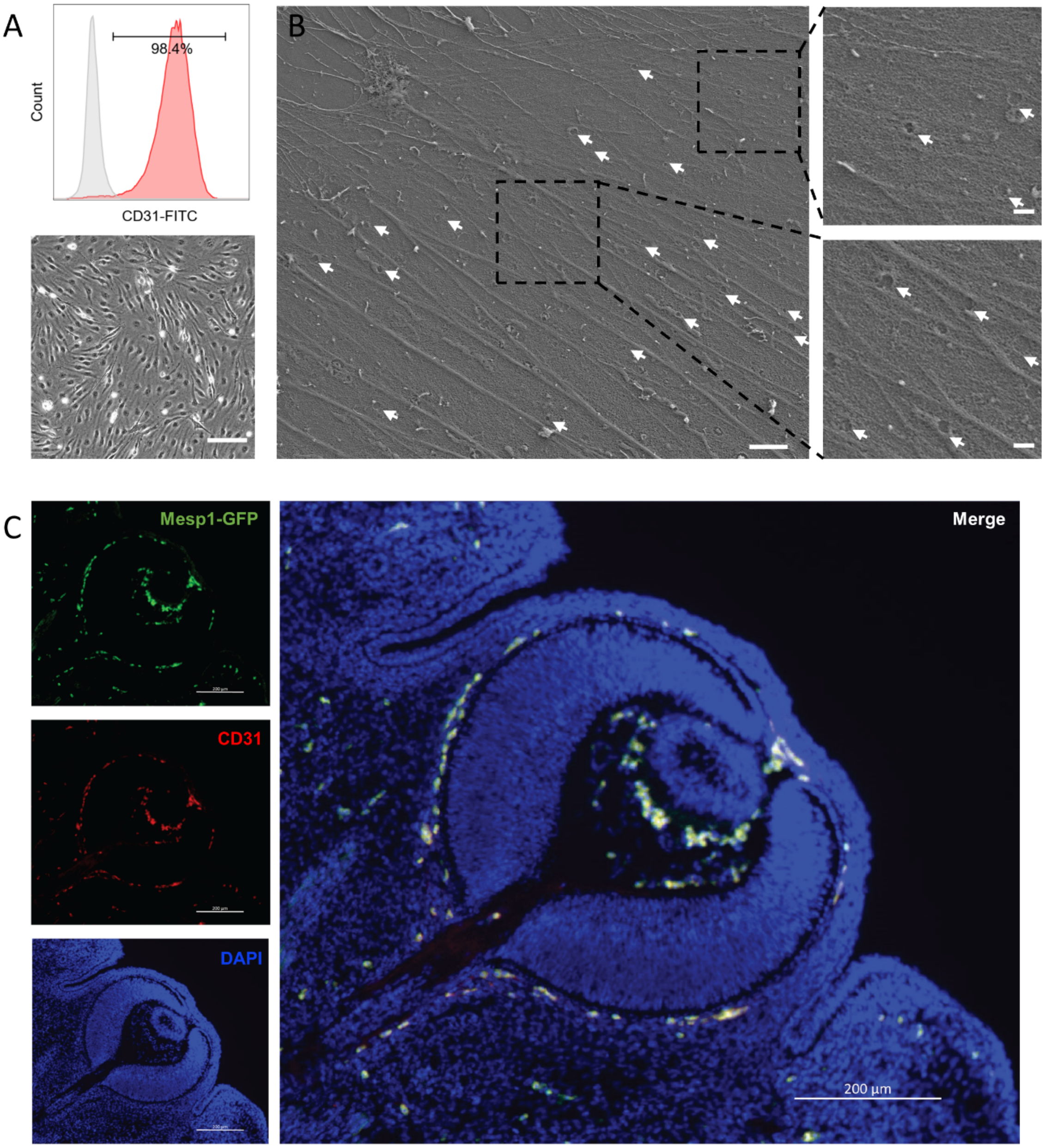

Supplemental Figure 2. Calculation of the Normalized Choroid Thickness (NCT)

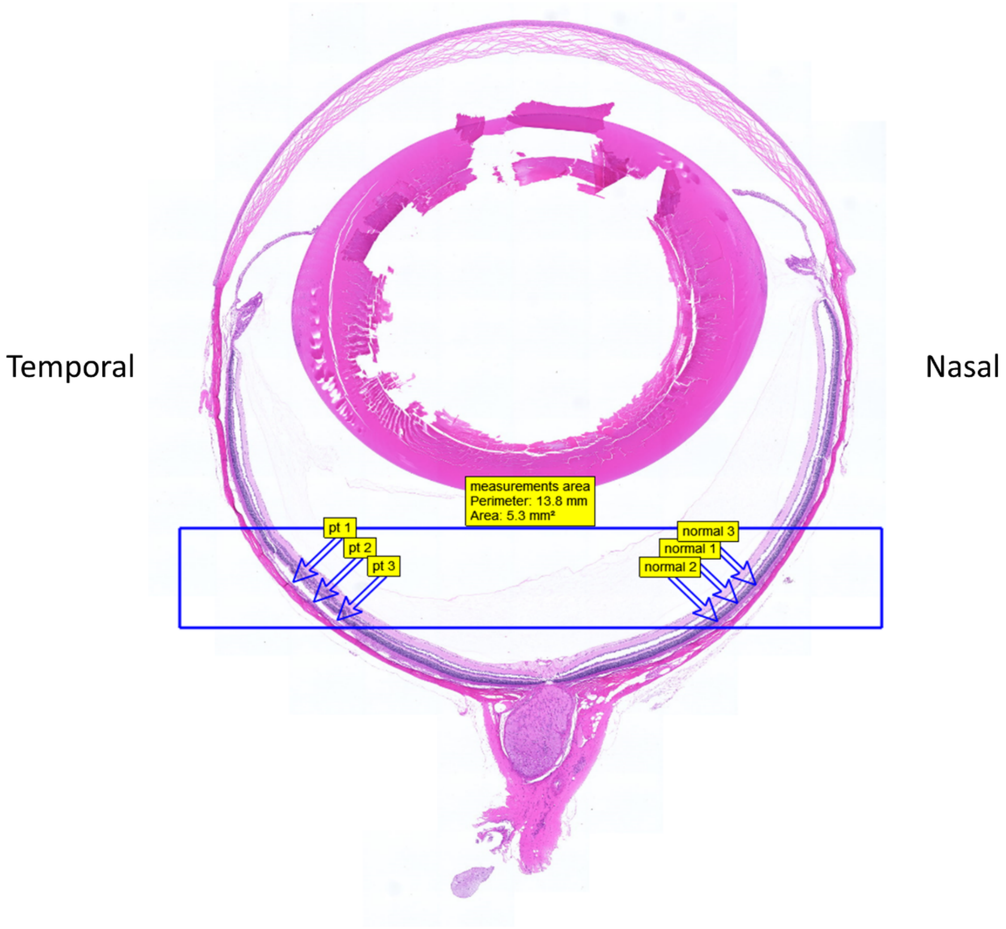

Three points were randomly chosen at location of injection (pt 1, pt2, pt3), and the opposite side where the choroid is normal(normal 1, normal 2, normal 3)

The Normalized Choroid Thickness =

$$\frac{pt1 + pt2 + \ pt3}{normal\ 1 + normal\ 2 + normal\ 3}$$

Supplemental Figure 3. Calculation of the Vessel Junction Density

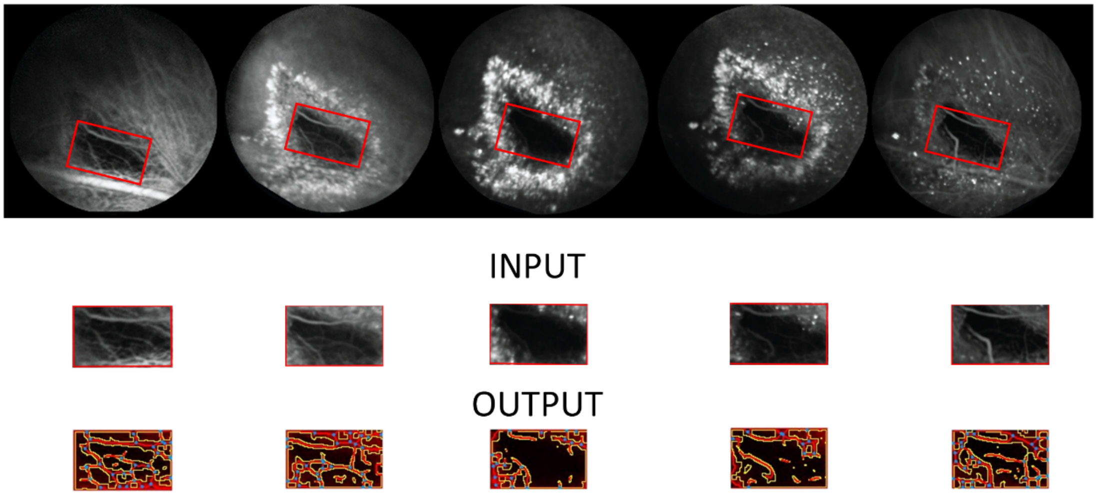

Explant area	Vessels area	Vessels percentage area	Total Number of Junctions	Junctions density	Total Vessels Length	Average Vessels Length	Total Number of End Points	Mean E Lacunarity
8165	3842	47.0545	31	0.00379669	852.7422	284.2474	15	0.071843
7797	3422	43.88868	29	0.00371938	815.4285	271.8095	16	0.106339
7910	2404	30.39191	18	0.0022756	592.3919	148.098	17	0.575613
8023	2695	33.59093	10	0.00124642	651.1615	72.35127	19	0.280754
7797	3352	42.99089	31	0.00397589	838.0854	139.6809	15	0.145883

Supplemental Figure 4. Single-cell analysis of hPSC-EC before and after co-culture with rat choroid for 48 hours

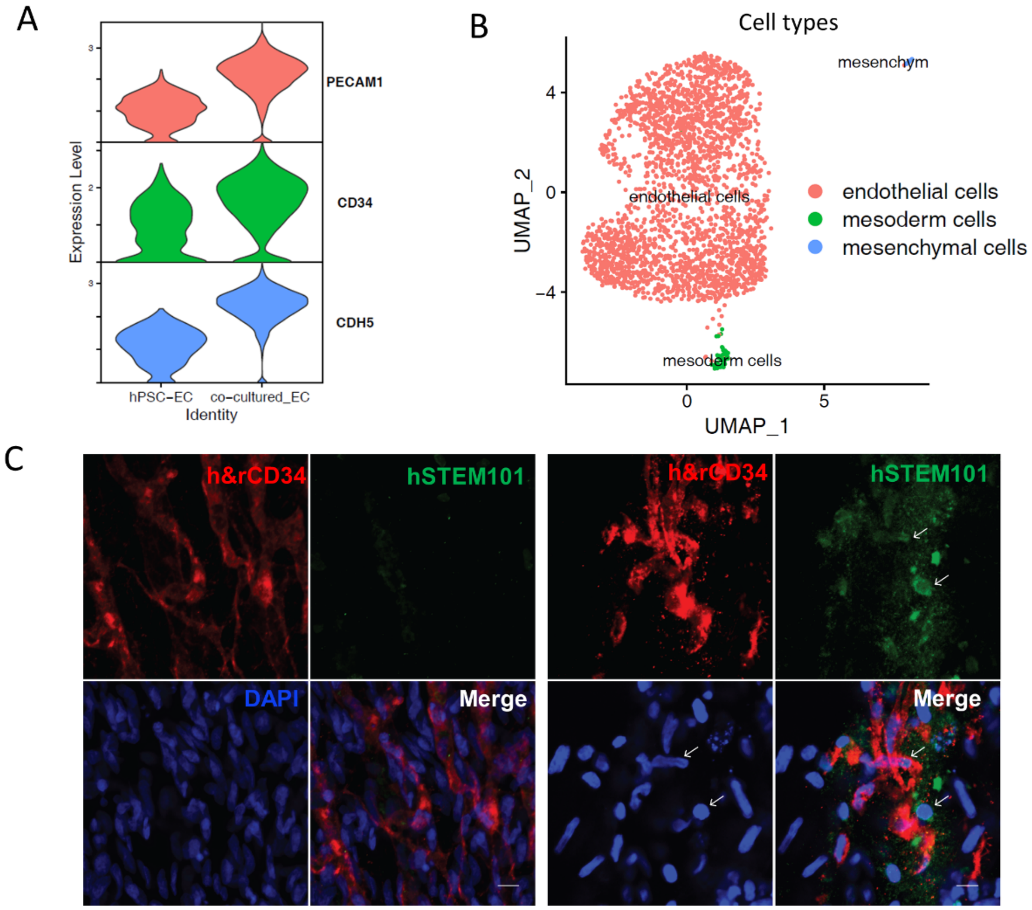

Supplemental Figure 5. scRNA-seq analysis of in vivo and ex vivo cultured ischemic rat choroid

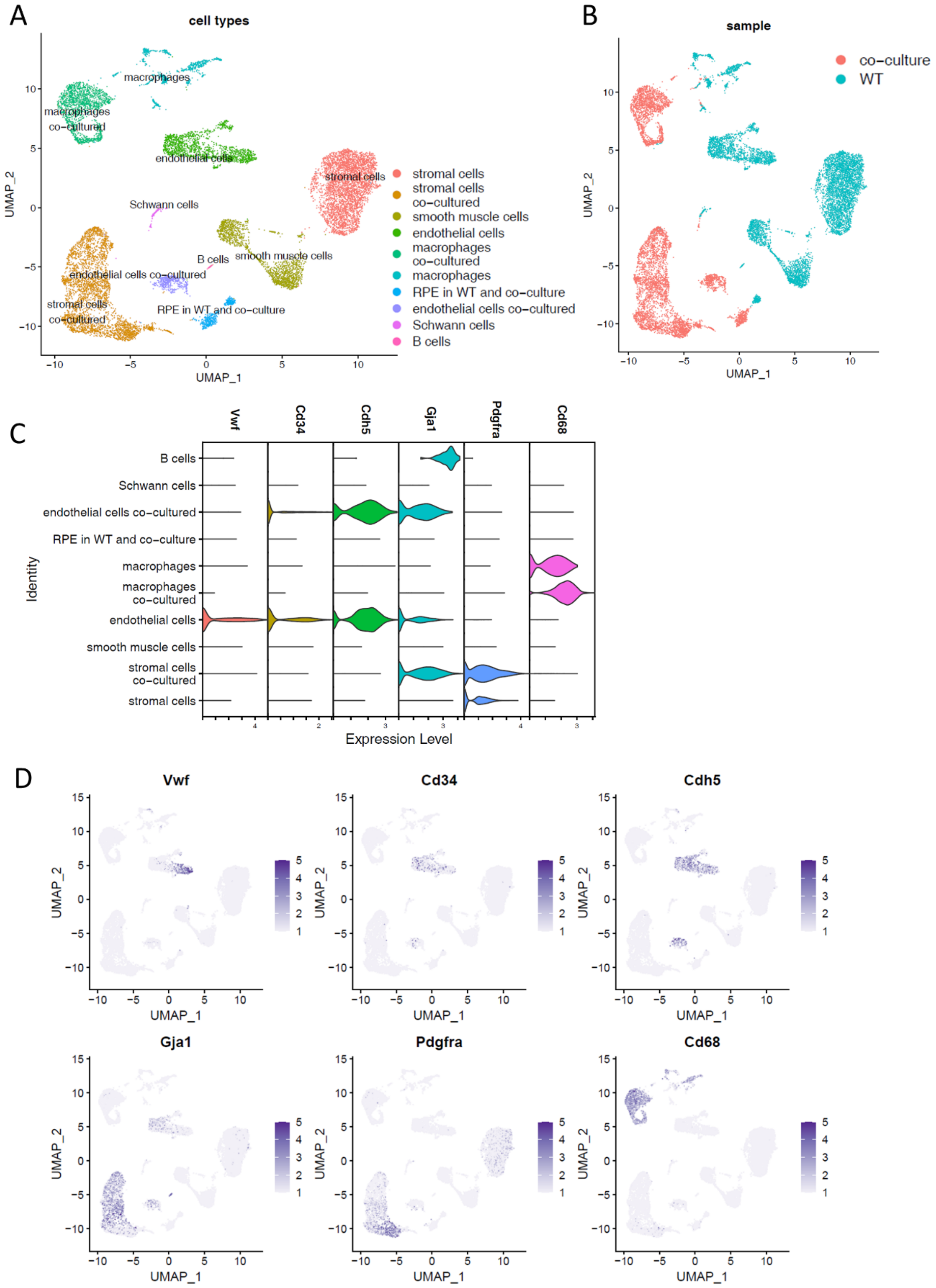

Supplemental Figure 6. Illustration of suprachoroidal injection of hPSC-ECs.

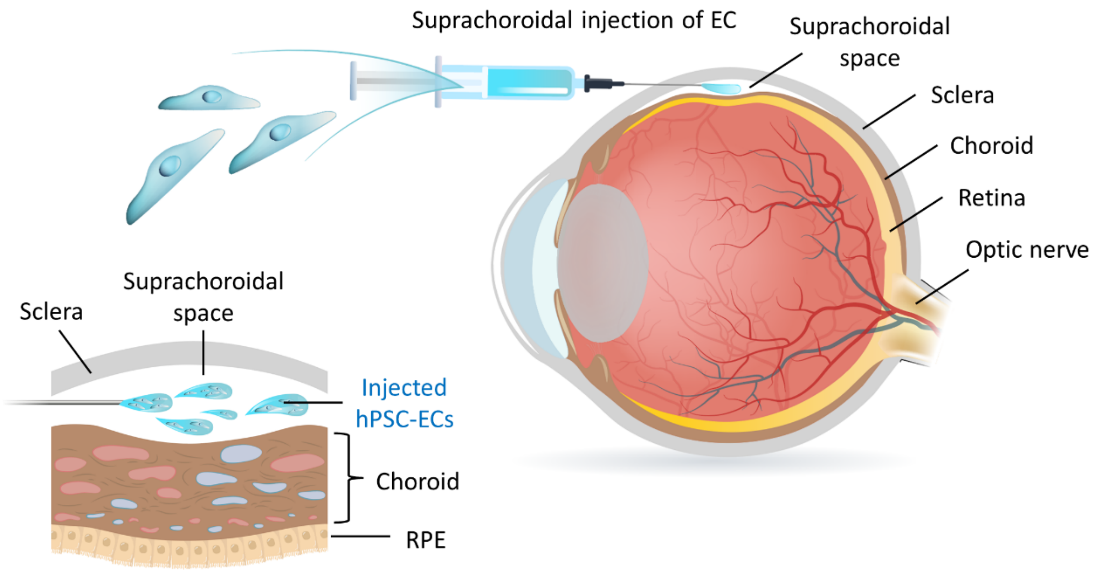

Supplemental Figure 7. Immunofluorescence images of the choroid flatmount and cryosections after H1-EC or iPSC-EC transplantation

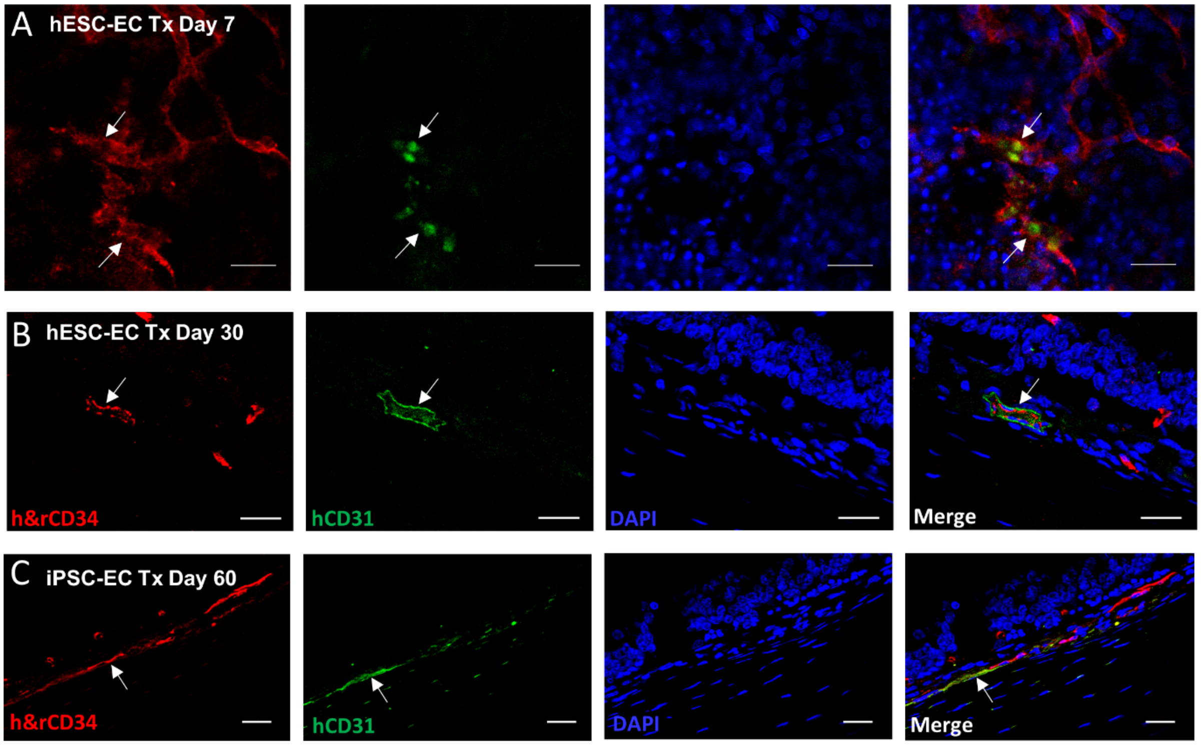

Supplemental Figure 8. Transplantation of iPSC-EC, EC culture media and MSC into CI model rats

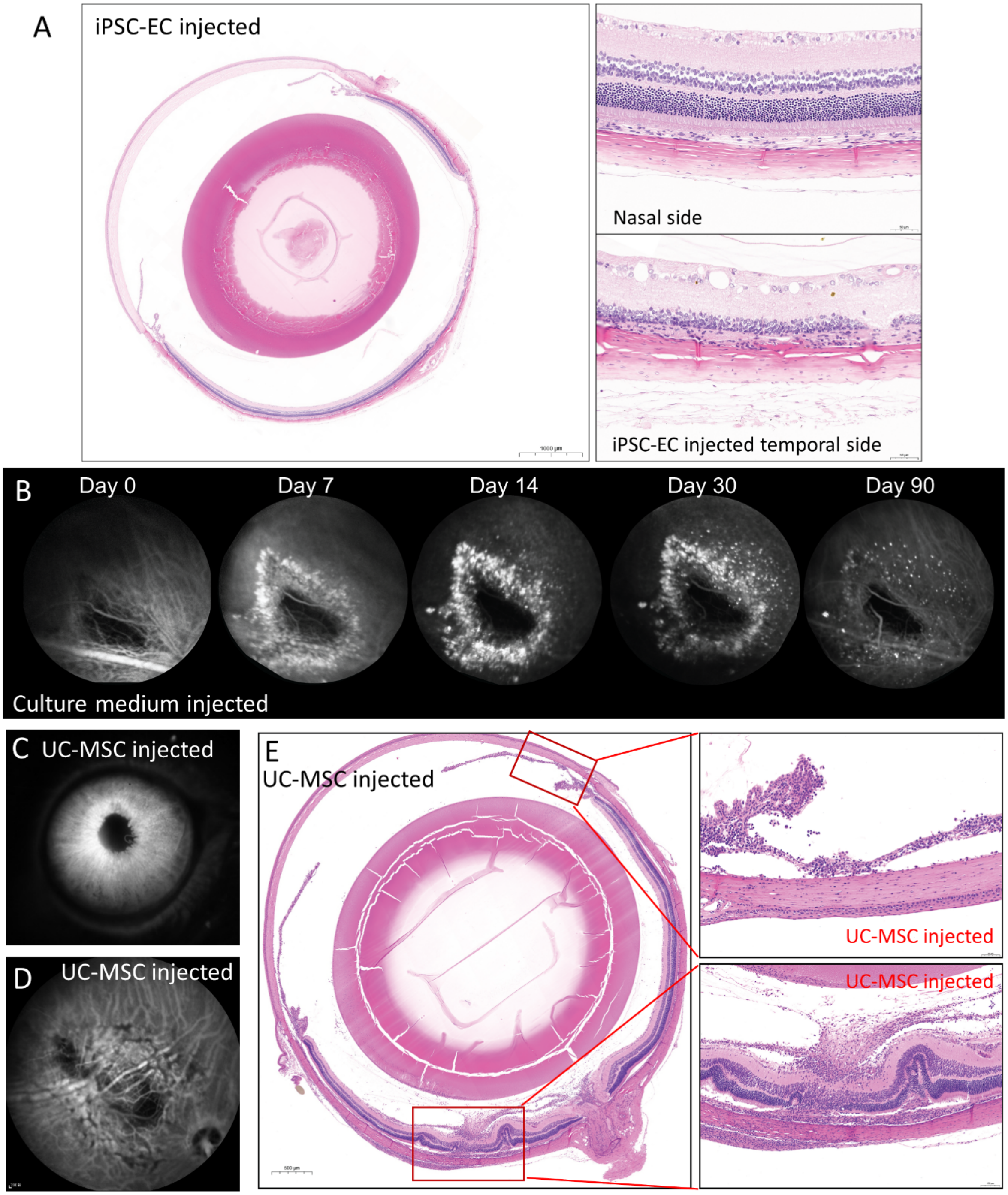

Supplemental Figure Legends

Supplemental figure 1. Analysis of ECs differentiated from Mesp1 progenitor cells.

A. Flow cytometry analysis of H1-ECs stained with CD31-FITC showed a purity of 98.4% (left). Light microscopy of the H1-EC. Scale bar = 25 µm.

B. Scan Electron Microscopy images of in vitro differentiated hPSC-EC. Arrow indicated fenestrae. The diameters of these fenestrations range from 100 nm to 200 nm. Scale Bar: Left = 2 µm. Right = 500 nm.

C. Immunofluorescence of eye section from E13.5 transgenic mouse embryos. Mesp1 descendent cells (green), CD31(red), and DAPI (blue). Note that choroid blood vessels (labeled in green and red) were derived from Mesp1 cells. Scale bar = 200 µm.

Supplemental figure 2. Calculation of the normalized choroid thickness (NCT) using H&E staining images.

Calculation of the normalized choroid thickness (NCT) using H&E staining images.

Supplemental Figure 3. Calculation of the Vessel Junction Density

Sample assessment of the vessel junction density using FA/ICGA images.

Supplemental Figure 4. Single-cell analysis of hPSC-EC before and after co-culture with rat choroid for 48 hours

A. Violin plot showing normalized expression levels of endothelial marker genes.

B. UMAP plot showing human cells in co-culture sample are mostly endothelial cells.

C. Immunofluorescence of rat choroid and choroid co-cultured with hPSC-EC for 48 hours. r&hCD34 (red), hSTEM101 (green), DAPI (blue); (n = 4, Scale bar = 20 µm.).

Supplemental Figure 5. scRNA-seq analysis of in vivo and ex vivo cultured ischemic rat choroid

A. UMAP plot showing annotated cell types in WT and co-culture samples.

B. Sample information projected into the same UMAP plot.

C. Violin plot showing normalized expression of representative genes.

D. UMAP plot showing normalized expression of representative genes.

Supplemental Figure 6. Illustration of suprachoroidal injection of hPSC-ECs.

A 2 mm cut was made to the temporal conjunctiva to expose the sclera. 1mL of $5{\times}10^4$ hPSC-EC was injected with a microliter syringe (Model 1701 RN SYR, Hamilton, NV, US) equipped with a 33G needle into the suprachoroidal space. The needle was inserted with a minimal angle parallel to the optic nerve 2 mm lateral to the limbus, then hPSC-EC was injected after the needle tip was 2 mm deep. (RPE: retinal pigment epithelium).

Supplemental Figure 7. Immunofluorescence images of the choroid flatmount and cryosections after H1-EC or iPSC-EC transplantation

A. Representative IF flatmount images of the choroid CI model transplanted with H1-EC 7 days post-transplantation. Scale bar = 20 µm.

B. Representative IF cryosection images of the choroid CI model transplanted with H1-EC 30 days post-transplantation. Scale bar = 20 µm.

C. Representative IF cryosection images of the choroid CI model transplanted with iPSC-EC 60 days post-transplantation. Scale bar = 20 µm.

Supplemental figure 8. Transplantation of iPSC-EC, EC culture media, and MSC into the rat CI model.

A. Representative H&E staining of iPSC-EC transplanted eyes on Day 60 post-transplantation. Left: Scale bar = 1000 μm, Right: Scale bar = 50 µm.

B. Representative FA/ICG images of a CI model injected with EC culture media 0, 7, 14, and 30 days post-procedure. Vessel density in the ischemia area decreased gradually.

C. Pathological changes induced by MSC transplantation into rat CI model: FA/ICGA images showing iris was posterior synechia and pupil failed to respond to dilation drops.

D. Disturbance of the retinal structure.

E. H&E staining showing inflammatory cell infiltration on the surface of the retina, vitreous cavity, and anterior chamber, and synechia of iris was also detected. Scale bar = 500 µm, Right: Scale bar = 50 µm.

Supplemental Table 1

Table S1. Primers for Q-PCR

Primer sequence	Reference	Tm
Primer[72]: PECAM1 Forward: CCAAGGTGGGATCGTGAGG	72	68°C
Primer: PECAM1 Reverse: TCGGAAGGATAAAACGCGGTC	72	67°C
Primer: CA4 Forward: TGGTCCGACTTGCCATATAAGG	72	67°C
Primer: CA4 Reverse: CTCTTTCACATTCCTCGATGTCC	72	65°C
Primer: RGCC Forward: CGCCACTTCCACTACGAGG	72	68°C
Primer: RGCC Reverse: CAGCAATGAAGGCTTCTAGCTC	72	66°C
Primer: PLVAP Forward: GCTGCTGGTATTACCTGCG	72	66°C
Primer: PLVAP Reverse: GCCATAGACCATGAAGAGCAC	72	66°C

Supplemental Files

1. Supplemental Video 1: Z-stack movie of IF images of rat choroid and hPSC-EC co-culture for 48 hours. hPSC-EC appeared to reconstruct the choroid vessels in two ways: hPSC-EC (green) could integrate into the damaged choroid vessels (red), or form new vessels by themselves.

2. Supplemental Video 2: Z-stack movie of IF images of choroid flatmount 90 days after hPSC-EC transplantation. hPSC-ECs (green) integrated into rat choroid vessels (red).
